# Long-acting or extended-release antiretroviral products for HIV treatment and prevention in infants, children, adolescents, and pregnant and breastfeeding women: knowledge gaps and research priorities

**DOI:** 10.1016/S2352-3018(19)30147-X

**Published:** 2019-07-12

**Authors:** Sharon Nachman, Claire L Townsend, Elaine J Abrams, Moherndren Archary, Edmund Capparelli, Polly Clayden, Shahin Lockman, Patrick Jean-Philippe, Kenneth Mayer, Mark Mirochnick, Jane McKenzie-White, Kimberly Struble, Heather Watts, Charles Flexner

**Affiliations:** a Health Sciences Center, SUNY Stony Brook, Pediatrics, New York, NY, USA; b Johns Hopkins University, Baltimore, MD, USA; c ICAP at Columbia, Mailman School of Public Health, Columbia University, New York, NY, USA; d Vagelos College of Physicians & Surgeons, Columbia University, New York, NY, USA; e University of Kwa Zulu Natal, Durban, South Africa; f Skaggs School of Pharmacy and Pharmaceutical Sciences, University of California, San Diego, CA, USA; g HIV i-Base, London, UK; h Botswana Harvard AIDS Institute Partnership, Gaborone, Botswana; i Department of Immunology and Infectious Diseases, Harvard TH Chan School of Public Health, Boston, MA, USA; j Harvard Medical School, Boston, MA, USA; k Division of Infectious Diseases, Brigham and Women's Hospital, Boston, MA, USA; l Maternal Adolescent Pediatric Research Branch, DAIDS/NIAID/NIH, Rockville, MD, USA; m School of Medicine, Harvard University, Cambridge, MA, USA; n Boston University School of Medicine, Boston, MA, USA; o Food and Drug Administration, Silver Spring, MD, USA; p Office of the Global AIDS Coordinator and Health Diplomacy, Washington, DC, USA

## Abstract

Antiretroviral agents with long-acting properties have potential to improve treatment outcomes substantially for people living with HIV. In November 2017, the Long acting/Extended Release Antiretroviral Resource Program (LEAP) convened a workshop with the aim of shaping the research agenda and promoting early development of long-acting or extended release products for key populations: pregnant and lactating women, children aged up to 10 years, and adolescents aged 10–19 years. Goals included strategies and principles to ensure that the needs of children, adolescents, and pregnant and lactating women are considered when developing long-acting formulations. Research should focus not only on how best to transition long-acting products to these populations, but also on early engagement across sectors and among stakeholders. A parallel rather than sequential approach is needed when establishing adult, adolescent, and paediatric clinical trials and seeking regulatory approval. Pregnant and lactating women should be included in adult clinical trials. Adolescent-friendly trial design is needed to improve recruitment and retention of young people.

## Introduction

In 2017, an estimated 10·1 million women and 1·8 million children younger than 15 years worldwide were living with HIV.^1^ Women continue to account for a disproportionate percentage of new HIV infections among adults (aged 15 years and older) in sub-Saharan Africa: this population represented 59% of the 18 million new adult HIV infections in 2017. Meanwhile, the number of adolescents (aged 10–19 as defined by WHO) living with HIV has increased.^2^ Daily oral antiretroviral therapy (ART) has had a substantial impact on treatment and prevention of HIV, but inadequate or incomplete adherence, leading to treatment failure and drug resistance, are major obstacles. In the USA, only 54% of young people who begin ART achieve viral suppression, and only 6% of young people maintain suppression in the long term.^3^ Adolescents often have adherence challenges, which puts both their health and the health of their sexual partners at risk.^4^, ^5^

Long-acting or extended-release (herein referred to as long-acting) nanoproducts of antiretroviral therapies and other HIV related treatments (such as monoclonal antibodies) are given on a monthly or less frequent basis and have the potential to play a key part in HIV treatment and prevention, particularly in populations where poor adherence hinders successful treatment.^6^ As with any novel drug, long-acting antiretroviral agents must undergo rigorous assessment of safety and efficacy. Some products might have additional safety considerations compared with their oral counterparts.^6^, ^7^, ^8^ Several long-acting products are in phase 1–3 trials for either treatment (NCT02951052) or prevention (HTPN084) of HIV-1 infection, including cabotegravir and rilpivirine, and other treatments such as broadly neutralising anti-HIV-1 monoclonal antibodies.

Historically, new HIV drugs and products have taken time to become available for children and pregnant and breastfeeding women. Concerns about teratogenicity of dolutegravir highlight the importance of increasing participation of pregnant woman in clinical research.^9^, ^10^, ^11^ Pregnant and lactating women are often excluded from clinical trials, and studies in children are typically delayed until phase 3 trials in adults have been completed.^12^, ^13^ Mechanisms to include these populations earlier in the drug development process and in parallel with studies in non-pregnant adults are urgently needed to achieve concurrent development of paediatric and adult products.^14^, ^15^ With clinical investigations of long-acting antiretroviral products in adults now proceeding, plans for including paediatric, adolescent, and pregnant and breastfeeding populations in clinical research into these agents should proceed without delay.

The Long Acting/Extended Release Antiretroviral Resource Program (LEAP) convened a workshop in Rockville, MD, USA, in November 2017, to generate discussion among key experts and stakeholders around long-acting products for pregnant and lactating women, infants, children, and adolescents.^16^ A complete description of the workshop and list of participants is provided in the appendix (p 1). Key questions that provided focus for presentations and discussion are presented in panel 1. This paper summarises the key principles discussed at the workshop and presents several research priorities identified and agreed upon by workshop participants to facilitate the development of long-acting products for both treatment and prevention in infants, children, adolescents, and pregnant and lactating women.

Panel 1 **Workshop discussion questions**

**Topic 1**

• Should long-acting, extended release formulations for children: should they be the same as for adults?
• Discuss the advantages and disadvantages of trying to develop unique formulations for paediatric applications (eg, subcutaneous dosing rather than intramuscular)
• Can and should implants and alternative drug delivery technologies be developed for children?

**Topic 2**

• What to do when a woman becomes pregnant?
• How would recommendations differ for a woman who becomes pregnant while on long-acting, extended release drugs for treatment or prevention, versus managing a pregnant woman who asks to initiate a long-acting, extended release regimen?

**Topic 3**

• What are the main challenges in enrolling and retaining adolescents in clinical trials for HIV treatment and prevention?
• What are the possible solutions to improve retention?

**Topic 4**

• What are the major regulatory and formulation approval challenges related to these three key populations?
• Are there easy-to-implement, innovative approaches to speed approval?

## Discussions

### Products for pregnant and breastfeeding women

Long-acting products could provide substantial benefits over the standard of care for women in the ante-partum, pregnancy, and post-partum periods (including while breastfeeding or at risk for new pregnancies), including improved adherence and viral suppression during times when adherence might be reduced.^17^, ^18^, ^19^, ^20^ Such products have been widely used for contraception and their acceptance is well documented.^21^ Long-acting products might also be beneficial for prevention of HIV at these times when women are at increased risk of HIV acquisition and, if they seroconvert, at increased risk of vertical transmission.^22^, ^23^, ^24^ It is also important to consider that many women of childbearing potential might become pregnant while taking long-acting products (for treatment or prevention), which necessitates a thorough understanding of safety and efficacy of these drugs during pregnancy.

Pregnant and lactating women are excluded from many clinical trials of novel antiretroviral agents and other therapies, leading to a paucity of data to support or discourage the use of these drugs throughout pregnancy and lactation.^25^ At all study phases, clinical trials of antiretroviral drugs have reported high pregnancy rates among enrolled women, despite inclusion criteria specifying use of contraception. Fetal exposure is evidently unavoidable during clinical trials that include women of childbearing potential.^26^, ^27^ Structures are needed to collect post-marketing safety and efficacy data of sufficient quality and quantity to assess outcomes from exposures in pregnancy.

In the absence of specific studies to obtain pregnancy safety data before drug licensure, such data could be obtained from registries and observational studies after a drug is marketed. However, detection of safety issues associated with drug exposure during pregnancy or lactation are thereby delayed while these populations are exposed to potentially inadequate or harmful treatments.

In addition to the possible risks of fetal exposure to new drugs, we must consider the risks of withholding potentially more convenient or efficacious drugs, or newer therapies with less toxicity from pregnant and lactating women with relevant conditions, because of paucity of data.^28^ Exclusion of this population from clinical trials could also delay country-level introduction of drugs, as new treatments cannot and should not be recommended for the general population until safety concerns and dosing in pregnant women are established. Therefore, inclusion of pregnant and lactating women in clinical trials of new therapies should occur when preliminary safety data are available, and early in phase 2 and 3 drug development programmes, provided that the prerequisite non-clinical data are complete and acceptable.

Reproductive toxicity studies should be done early on, to provide the risk data required for the design of clinical studies. Ex vivo placental perfusion models can be helpful in providing a preliminary understanding of the kinetics of placental transfer of novel long-acting agents, but data from humans are necessary to accurately describe placental transfer kinetics.

Two main scenarios exist in which long-acting products could be studied in pregnant and lactating women: when unintended or unplanned pregnancies occur in women participating in clinical trials, and active prospective enrolment in clinical trials. Eventually, high-quality postmarketing surveillance studies could also provide important data. Ongoing and future studies of investigational agents in non-pregnant women should allow those becoming pregnant during the trial to remain in the study and on their assigned study drug, with collection of key safety and efficacy data (for mothers and infants) in a systematic and structured manner, following additional informed consent. This approach should be the norm, unless there are specific reasons against inclusion of pregnant women, such as preclinical data indicating a safety concern for mothers or fetuses or pharmacokinetic modelling showing inadequate exposures in pregnant women. Therefore, pregnant women could feasibly be involved in clinical trials, providing specific criteria are met;^29^ provisions should be made for additional safety monitoring, and data on fetal exposure and pregnancy outcome should be collected. Similar to US Department of Health and Health Services guidance, standardised monitoring is also recommended by the WHO Paediatric Antiretroviral Working Group and other international groups.^30^, ^31^ This position is particularly important for therapies likely to be used by many women of childbearing potential and is supported by federal regulations, which allow inclusion of pregnant women and fetuses in research as long as various criteria are met.^32^ Pharmacokinetic studies in pregnant and lactating women are needed to measure effects of physiological changes associated with pregnancy on drug absorption, distribution, biotransformation, and elimination;^33^ to inform the need for dose adjustments; and to understand in-utero exposure.

Consideration should be given to dedicated pharmacokinetic and phase 2 studies during pregnancy, once phase 1 and 2 data from non-pregnant adults are available. Consideration should also be given to phase 3 studies in pregnant women if the likelihood of safety concerns is low on the basis of preclinical or pharmacokinetic modelling data. For initial pharmacokinetic studies of long-acting products in pregnancy, these drugs could be added to a suppressive regimen to provide added protection from transmission during delivery and breastfeeding. Conversely, if enough women in the phase 3 trial became pregnant and remained on study drug, and if the data collected could help establish pharmacokinetics and dosing in pregnancy, then an additional study might not be needed. However, to rule out less frequent safety issues in pregnancy (which would require larger sample sizes), an observational study after licensure would be needed.

A standardised protocol for women who become pregnant during a trial should also be developed and made available, in an open forum, for use by all. This protocol could be adapted for different therapeutic agents and would outline the minimum pharmacokinetic, pregnancy outcome, and maternal and infant safety and toxicity monitoring required for all women who become pregnant during a study. In addition, infant follow-up data, up to at least 1 year of age, or through cessation of breastfeeding would be collected. Similarly, standard protocols for establishing postmarketing surveillance of pregnancy outcomes and safety and efficacy in the context of the roll-out of new products should be created and implemented.

An online database could be established or an existing pregnancy database used to capture data across different studies and data on inclusion of pregnant and lactating women in research into long-acting products. This database could include a summary of preclinical reproductive toxicity of investigational agents; a record of the number of pregnant and lactating women being actively enrolled in trials and number becoming pregnant during a trial; details of cessation or continuation of study drugs; and a protocol for infant follow-up.

Although some medical care providers and pregnant and lactating women might be more cautious in accepting a new treatment, these therapies are often used in clinical practice, once licensed and available in-country. Acceptability studies in people living with HIV for novel drug-device combinations are suggested but not required and could be nested within clinical trials.

### In-utero exposure

Establishing the fetal safety profile of a product is difficult before marketing, as sufficient exposure data across trimesters are needed. Women becoming pregnant on long-acting products during clinical trials are advised to stop the drugs early in pregnancy, limiting the extent of fetal exposure. However, with potentially longer drug half-life, more inclusive trial designs, and more widespread use of long-acting products in late pregnancy, newborn exposure data collection will be possible. Products given to pregnant women might cross the placenta into the fetal compartment; thus neonatal washout studies describing elimination from the newborn after in-utero exposure can provide valuable information on drug elimination in the first week of life. Such data can then be used in modelling studies to inform selection of initial doses in newborn prevention and treatment studies. Protocols should be developed that collect data on newborn exposure to long-acting products used for prevention of HIV transmission during pregnancy, at delivery, and post partum at the earliest opportunity, to support the continued use of these products in pregnant and lactating women, and ultimately their use in newborns.

### Neonatal prophylaxis and treatment

The risk of perinatal HIV acquisition in infants born to women with HIV can be substantially reduced with administration of antiretroviral prophylaxis to these infants.^34^ Timing of length of therapy and treatments used is dependent on maternal risk factors and length of breastfeeding. Similarly, human studies in lactating women and their infants are needed to describe the kinetics of breastmilk transfer of long-acting agents, even if an oral counterpart is already approved. Establishment of safety and efficacy via administration of a single injection or microneedle patch to newborn babies has the potential to simplify neonatal prophylaxis greatly and to act as a bridge to neonatal treatment. Safety of long-acting products passing through breastmilk must also be investigated.

### Treatment of children across age and weight bands

Accelerating the development of paediatric (outside of the neonatal period) antiretroviral drug products has been the focus of various international initiatives,^14^, ^35^ and this need for acceleration was reflected in the views of workshop participants in relation to long-acting products. Providing there was a prospect of benefit and no known safety concerns, products should be developed with the whole age spectrum in mind and with the aim of achieving concurrent licensure for adults, adolescents, and children. To accomplish this goal, protocols for trials of long-acting products in children should be developed by experts in the field, alongside development of studies in adults, with paediatric studies initiated as soon as both early safety data and prospect of benefit from adult trials are available. As has been recommended for studies of oral antiretroviral drugs, harmonisation of long-acting products across weight bands is crucial, with paediatric clinical trials designed to align with WHO weight bands when possible.^11^ Furthermore, in the absence of known safety concerns that could affect willingness to give the drug to younger children, paediatric clinical trials should simultaneously enrol all paediatric age and weight cohorts rather than sequentially enrolling cohorts of decreasing age. One of the major discussion points at the workshop was the issue of dosing during rapid growth periods. Plans for dose adjustment during rapid growth periods must be developed.

For long-acting products to be effective in the paediatric population, qualitative studies should be done early in development to understand the attitudes of parents, care providers, and children of different ages and across different cultures to gauge interest in novel delivery systems. The inclusion of such studies within the paediatric clinical development plan will ensure that data are available to inform formulation development, help identify priorities and understand the likely market demand, and support more effective implementation within each target population.

### Treatment and prevention in adolescents

Research into the safety, efficacy, and acceptability of novel drug delivery systems has been suggested as one of the top five priorities relating to the treatment of adolescents living with HIV.^36^ However, despite changes in the regulatory environment that have facilitated clinical trials in children and adolescents, inequities continue to exist and trials in adolescents living with HIV remain few.^13^ Although additional considerations might be required when doing research in adolescents, to ensure that trial sites are adolescent-friendly, a balance is needed between protecting patients from harm and ensuring adequate representation of adolescents in research, leading to better access to much-needed drugs.^37^ Research is also needed into the acceptability of injectable products for adolescents.^38^ Long-acting products might provide substantial benefits during adolescence (a period when adherence is often problematic) particularly as simplified regimens with reduced frequency of dosing are developed.

Adolescent-specific dosing should also consider weight and not just age for enrolment. Pharmacokinetics in adolescents can be evaluated in adult phase 3 clinical trials, rather than in concurrent adolescent trials. Workshop participants suggested that if safety concerns are unlikely, the upper limit of target drug concentrations (area under curve and trough concentrations) could be widened for adolescents weighing 25–35 kg. Typically, adult doses and formulations are used in adolescent trials. Workshop participants agreed that adolescents must be included in adult phase 3 clinical trials of novel products to inform future use in this age group.

Workshop participants identified strategies to facilitate recruitment and retention of adolescents in clinical trials (panel 2), and proposed that internationally agreed standards for adolescent clinical trials should be developed, drawing on existing ethical benchmarks for adolescent research.^39^

Panel 2 **Suggested approaches for facilitating recruitment and retention of adolescents in clinical trials**

**Recruitment**

• Recruit youth-friendly and peer staff.
• Identify locations where adolescents can be approached.
• Tap into social network and opinion leaders.

**Retention**

• Ensure regular contact with participants.
• Take the intervention to the participant if possible.
• Provide youth-friendly space and hours.
• Develop an outreach package specific to the trial and population.

**General considerations**

• Ensure good links to other services, such as sexual health and HIV treatment services.
• Ensure that providers (adult and paediatric) are educated about long-acting, extended release formulations and comfortable with the trial intervention.
• Provide funding incentives for trial sites that adhere to youth-friendly practices.

### The future of long-acting products

Ideal requirements of long-acting products would include low volume and number of injections, high barrier to resistance, and product transport and storage conditions compatible with use in low-income and middle-income settings (ie, avoiding the need for refrigeration or freezing). Products and delivery systems that are feasible, acceptable, and affordable in such settings are key, given the location and size of the market for paediatric HIV treatment. Given the limited availability of oral antiretroviral products for children (few fixed-dose combinations, paucity of chewable or dispersible products, need for refrigeration or other supply chain issues), availability of long-acting products for all paediatric weight bands is crucial. The need for an oral lead-in for long-acting antiretrovirals could delay the introduction of injectable long-acting products for paediatric use; products that do not require an oral lead-in are therefore desirable.

Novel drugs with their associated delivery systems might improve treatment options for all, particularly if frequency of dosing could be further reduced, active pharmaceutical ingredients were combined into a single formulation, or injections were replaced with alternative delivery systems such as transdermal microneedle patches or implants.^40^ Although research into such products continues, the development of paediatric fixed-dose, once-daily oral products remains a priority for paediatric HIV treatment.^41^

Early engagement between drug manufacturers and other stakeholders is important for the successful development of long-acting products for use across different population groups. Discussions with regulatory agencies will clarify the data required for licensing, and early engagement with communities will ensure that the needs of children, adolescents, and pregnant and lactating women are better understood and considered early in the development process.

Given the differences between oral antiretrovirals and long-acting products in terms of both administration route and dose frequency, the introduction of these products into national treatment programmes must be carefully planned, with consistent messages across partners. Effective, centrally-led strategies must be in place before such products are launched, particularly in low-income and middle-income countries. This will require collaboration and coordination across sectors, from suppliers and international bodies to in-country health services, with appropriate training for clinic and community staff.

Collection and dissemination of lessons learned from long-acting products developed for other diseases such as antipsychotics (ie, aripiprazole, olanzapine, paliperidone), particularly on the practicalities of launching a new treatment approach and its uptake by clinicians, could help facilitate the introduction of long-acting antiretroviral products. Monitoring of uptake and adherence will be needed to learn from early adopters, to inform the introduction of these products in other settings, and to plan at a global level to avoid stockouts or wastage. Additional considerations will be required if long-acting products are used for HIV prevention, particularly in understanding people's motivations in seeking prevention and how this drives demand.

A harmonised approach would encourage engagement of innovator and generic companies by creating a more viable market, simplifying development in the long term and facilitating the introduction of long-acting antiretroviral treatment at the country level. These goals can only be achieved through coordinated action, with involvement of all key stakeholders from the start, and with alignment of donors to maximise support in the development and implementation phases. Good communication is needed across sectors to ensure that manufacturers are aware of the requirements for regulatory approval and to meet the needs of patients and providers regarding products and delivery systems.

### Post-meeting events

Experience with daily dolutegravir and its possible link to neural-tube defects in infants born to women taking it as part of standard of care, including at the time of conception^9^, ^10^, ^11^ has highlighted the need to enrol pregnant women in studies of novel products.^42^ The study is ongoing,^43^ and data from this and other investigations should provide more information to assess this therapy's use. The existence of this birth outcome surveillance project before and after the Botswana guidelines changed in 2016^44^ permitted this analysis to occur in a timely manner, allowing for recognition of a possible signal regarding a new agent.

## Conclusions

The research agenda can be tailored in various ways to generate data needed for regulatory approval of long-acting products for pregnant and lactating women, children of all ages, and adolescents (panel 3).

Panel 3 **Key recommendations for the development of long-acting or extended release formulations for neonates, children, adolescents, and pregnant and breastfeeding women**

**Pregnant and breastfeeding women**

• Studies of investigational agents in non-pregnant women should allow those becoming pregnant during the trial to remain in the study and on their assigned study drug, with additional informed consent, unless there is clear justification for exclusion.
• Pregnant and breastfeeding women should be considered eligible for active enrolment in clinical trials unless clear justification for exclusion exists.
• Additional monitoring of maternal, pregnancy, and infant outcomes should be undertaken for all women who become pregnant during a clinical trial and all pregnant and breastfeeding women actively enrolled in trials with a standard protocol.

**Neonates (birth to 4 weeks)**

• Neonatal washout studies are needed that describe the disappearance of drug present at birth after in-utero exposure, followed by dosing studies to describe safety and pharmacology in neonates and young infants to allow use of novel formulations for neonatal and young infant prophylaxis and early treatment.

**Children (4 weeks to 10 years)**

• Protocols for trials of long-acting, extended release formulations in children should be developed alongside adult trials, and paediatric studies should be initiated as soon as early safety data from adult trials is released.
• Paediatric clinical trials should simultaneously enrol all paediatric age and weight cohorts rather than sequentially enrolling cohorts of decreasing age or size.
• Long-acting formulations for children should be harmonised across weight bands.

**Adolescents (10–19 years)**

• Adolescents should be included in adult clinical trials of long-acting antiretroviral agents, or in separate trials proceeding nearby.
• Strategies to maximise recruitment and retention of adolescents in clinical trials should be employed.

**General recommendations**

• Acceptability studies should be done early in the development of long-acting formulations to understand the attitudes of care providers, children of different ages and their parents, and pregnant and breastfeeding women.
• Coordinated action involving all key stakeholders is required from the early stages of development to maximise the chances of success.

Pregnancy intentions should not be a barrier to enrolment of women of childbearing potential, and we encourage inclusion of pregnant and lactating women in all appropriate phase 3 clinical trials. Paediatric studies inclusive of all age groups should be planned early in development, and if possible should be carried out as soon as preliminary adult safety and efficacy data are available from phase 1 and 2 trials. Products should be applicable across all weight bands, including adults, to facilitate implementation, ensure market viability, and maximise the chance of success of long-acting therapeutic strategies. Efforts should be made to include adolescents in adult clinical trials or to establish adolescent-specific trials in parallel where appropriate. These studies should use a range of methods to maximise recruitment and retention of patients, including good community engagement and adolescent-friendly approaches. Studies are also needed to understand the acceptability and feasibility of long-acting products in all populations, particularly in settings with high HIV burden.

As we assess how best to study the use of long-acting products in children, adolescents, and pregnant and lactating women, and ultimately transition these new drug delivery products and systems into these populations, it is also worth considering which products would be best suited and of most benefit to specific populations. Although injectable products have the potential to improve treatment outcomes, particularly in groups with poor adherence, frequent injections could be a barrier in children. Delivery systems for long-acting products are needed that are less painful or require less frequent dosing—and are therefore more acceptable to children, adolescents, and their parents and care providers—such as implants and transdermal patches. New drugs and technological platforms are essential to support the development of long-acting treatment for infants, children, and adolescents. Removable microneedle patches have the potential to deliver long-acting antiretroviral agents without the pain associated with injections and could be applied at home or during a routine clinic visit. Long-acting oral preparations would also have potential, but systems developed to date are only in the preclinical phases. Research into alternative delivery mechanisms should therefore be encouraged.

Planning for novel treatment approaches involves actively engaging with patients and communities from an early stage to understand patient desires and motivations, and with donors, regulatory agencies, and pharmaceutical companies to ensure alignment and commitment of key stakeholders from the outset to maximise success. By working toward a research agenda that includes, from an early stage, infants, children, adolescents, and pregnant and lactating women, we can ensure that benefits of long-acting antiretroviral agents extend to these key populations without the delays that have occurred in the past.

## References

[1] UNAIDS (2018). UNAIDS Data 2018. https://www.unaids.org/sites/default/files/media_asset/unaids-data-2018_en.pdf.

[2] UNICEF (2016). For every child, end AIDS: seventh stocktaking report. https://www.unicef.org/publications/index_93427.html.

[3] Zanoni BC, Mayer KH (2014). The adolescent and young adult HIV cascade of care in the United States: exaggerated health disparities. AIDS Patient Care STDS.

[4] Kim SH, Gerver SM, Fidler S, Ward H (2014). Adherence to antiretroviral therapy in adolescents living with HIV: systematic review and meta-analysis. AIDS.

[5] Rudy BJ, Murphy DA, Harris DR (2009). Patient-related risks for nonadherence to antiretroviral therapy among HIV-infected youth in the United States: a study of prevalence and interactions. AIDS Patient Care STDS.

[6] Barnhart M (2017). Long-acting HIV treatment and prevention: closer to the threshold. Glob Health Sci Pract.

[7] Margolis DA, Boffito M (2015). Long-acting antiviral agents for HIV treatment. Curr Opin HIV AIDS.

[8] Jacobson JM, Flexner CW (2017). Universal antiretroviral regimens: thinking beyond one-pill-once-a-day. Curr Opin HIV AIDS.

[9] Zash R, Makhema J, Shapiro RL (2018). Neural-tube defects with dolutegravir treatment from the time of conception. N Engl J Med.

[10] AIDS*info* Statement on potential safety signal in infants born to women taking dolutegravir from the HHS antiretroviral guideline panels. https://aidsinfo.nih.gov/news/2094/statement-on-potential-safety-signal-in-infants-born-to-women-taking-dolutegravir-from-the-hhs-antiretroviral-guideline-panels.

[11] FDA (2018). FDA Drug Safety Communication: FDA to evaluate potential risk of neural tube birth defects with HIV medicine dolutegravir (Juluca, Tivicay, Triumeq). https://www.fda.gov/Drugs/DrugSafety/ucm608112.htm.

[12] Curno MJ, Rossi S, Hodges-Mameletzis I (2016). A systematic review of the inclusion (or exclusion) of women in HIV research: from clinical studies of antiretrovirals and vaccines to cure strategies. J Acquir Immune Defic Syndr.

[13] Joseph PD, Caldwell PH, Barnes EH, Craig JC (2017). Disease burden-research match? Registered trials in child health from low- and middle-income and high-income countries. J Paediatr Child Health.

[14] Penazzato M, Gnanashanmugam D, Rojo P (2017). Optimizing research to speed up availability of pediatric antiretroviral drugs and formulations. Clin Infect Dis.

[15] van der Graaf R, van der Zande IS, den Ruijter HM (2018). Fair inclusion of pregnant women in clinical trials: an integrated scientific and ethical approach. Trials.

[16] LEAP (2017). Long acting/extended release antiretroviral formulations for HIV treatment & prevention in children, adolescents, & pregnant women: knowledge gaps & approaches for development. https://www.longactinghiv.org/content/long-actingextended-release-antiretroviral-formulations-hiv-treatment-prevention-children.

[17] Bardeguez AD, Lindsey JC, Shannon M (2008). Adherence to antiretrovirals among US women during and after pregnancy. J Acquir Immune Defic Syndr.

[18] Mellins CA, Chu C, Malee K (2008). Adherence to antiretroviral treatment among pregnant and postpartum HIV-infected women. AIDS Care.

[19] Henegar CE, Westreich DJ, Maskew M (2015). Effect of pregnancy and the postpartum period on adherence to antiretroviral therapy among HIV-infected women established on treatment. J Acquir Immune Defic Syndr.

[20] Myer L, Phillips TK, Zerbe A (2018). Integration of postpartum healthcare services for HIV-infected women and their infants in South Africa: a randomised controlled trial. PLoS Med.

[21] Winner B, Peipert JF, Zhao Q (2012). Effectiveness of long-acting reversible contraception. N Engl J Med.

[22] Mugo NR, Heffron R, Donnell D (2011). Increased risk of HIV-1 transmission in pregnancy: a prospective study among African HIV-1-serodiscordant couples. AIDS.

[23] Drake AL, Wagner A, Richardson B, John-Stewart G (2014). Incident HIV during pregnancy and postpartum and risk of mother-to-child HIV transmission: a systematic review and meta-analysis. PLoS Med.

[24] Thomson KA, Hughes J, Baeten JM (2018). Increased risk of HIV acquisition among women throughout pregnancy and during the postpartum period: a prospective per-coital-act analysis among women with HIV-infected partners. J Infect Dis.

[25] Shields KE, Lyerly AD. Exclusion of pregnant women from industry-sponsored clinical trials. *Obstet Gynecol* **122:** 1077–81. 10.1097/AOG.0b013e3182a9ca67 24104789

[26] Serris A, Zoungrana J, Diallo M (2016). Getting pregnant in HIV clinical trials: women's choice and safety needs. The experience from the ANRS12169-2LADY and ANRS12286-MOBIDIP trials. HIV Clin Trials.

[27] Ssali A, Namukwaya S, Bufumbo L (2013). Pregnancy in HIV clinical trials in sub Saharan Africa: failure of consent or contraception?. PLoS One.

[28] Lyerly AD, Little MO, Faden R (2008). The second wave: toward responsible inclusion of pregnant women in research. Int J Fem Approaches Bioeth.

[29] US Department of Health and Human Service (2009). Code of federal regulations, title 45: public welfare, part 46: protection of human subjects. https://www.hhs.gov/ohrp/regulations-and-policy/regulations/45-cfr-46/index.html.

[30] Mastroianni AC, Faden R, Federman D (1994). Women and health research: ethical and legal issues of including women in clinical studies.

[31] Pariente G, Leibson T, Carls A (2016). Pregnancy-associated changes in pharmacokinetics: a systematic review. PLoS Med.

[32] HHS, FDA, CDER, CBER (2018). Pregnant women: scientific and ethical considerations for inclusion in clinical trials guidance for industry. https://www.fda.gov/downloads/Drugs/GuidanceComplianceRegulatoryInformation/Guidances/UCM603873.pdf.

[33] WHO (2016). Consolidated guidelines on the use of antiretroviral drugs for treating and preventing HIV infection: Recommendations for a public health approach—second edition. http://www.who.int/hiv/pub/arv/arv-2016/en/.

[34] UNAIDS, PEPFAR (2016). Start Free, Stay Free, AIDS Free: a super-fast-track framework for ending AIDS in children, adolescents and young women by 2020. https://free.unaids.org/.

[35] The International AIDS Society's Collaborative Initiative for Paediatric HIV Education and Research and the World Health Organization (2017). IAS and WHO identify the most critical research needs for infants, children and adolescents living with HIV. http://www.ias2017.org/Media-Centre/Press-Releases/ArticleID/90/IAS-and-WHO-identify-the-most-critical-research-needs-for-infants-children-and-adolescents-living-with-HIV.

[36] Nelson RM, Lewis LL, Struble K, Wood SF (2010). Ethical and regulatory considerations for the inclusion of adolescents in HIV biomedical prevention research. J Acquir Immune Defic Syndr.

[37] Bekker LG, Slack C, Lee S (2014). Ethical issues in adolescent HIV research in resource-limited countries. J Acquir Immune Defic Syndr.

[38] Flexner C (2018). Antiretroviral implants for treatment and prevention of HIV infection. Curr Opin HIV AIDS.

[39] Penazzato M, Lee J, Capparelli E (2015). Optimizing drugs to reach treatment targets for children and adolescents living with HIV. J Int AIDS Soc.

[40] Landovitz RJ, Kofron R, McCauley M (2016). The promise and pitfalls of long-acting injectable agents for HIV prevention. Curr Opin HIV AIDS.

[41] Kirtane AR, Abouzid O, Minahan D (2018). Development of an oral once-weekly drug delivery system for HIV antiretroviral therapy. Nat Commun.

[42] HHS (2018). Task force on research specific to pregnant women and lactating women. https://www.nichd.nih.gov/sites/default/files/2018-09/PRGLAC_Report.pdf.

[43] Zash R, Jacobson D, Mayondi G, et al. Dolutegravir/tenofovir/emtricitabine (DTG/TDF/FTC) started in pregnancy is as safe as efavirenz/tenofovir/emtricitabine (EFV/TDF/FTC) in nationwide birth outcomes surveillance in Botswana. IAS 2017. Paris, France; July 23–26, 2017. Abstract 5532.

[44] WHO Consolidated guidelines on the use of antiretroviral drugs for treating and preventing HIV infection. Recommendations for a public health approach—second edition. https://www.who.int/hiv/pub/arv/arv-2016/en/.

