# Supplementary material for: Long-acting or extended-release antiretroviral products for HIV treatment and prevention in infants, children, adolescents, and pregnant and breastfeeding women: knowledge gaps and research priorities
Source: Lancet HIV. 2019 Jul 12;6(8):e552–8. doi: 10.1016/S2352-3018(19)30147-X (PMC7152795; doi:10.1016/S2352-3018(19)30147-X)
Supplement: Supplementary appendix [file mmc1.pdf]

THE LANCET
HIV

Supplementary appendix

This appendix formed part of the original submission and has been peer reviewed. We post it as supplied by the authors.

Supplement to: Nachman S, Townsend CL, Abrams EJ, et al. Long-acting or extended-release antiretroviral products for HIV treatment and prevention in infants, children, adolescents, and pregnant and breastfeeding women: knowledge gaps and research priorities. *Lancet HIV* 2019; published online July 12. http://dx.doi.org/10.1016/S2352-3018(19)30147-X.

APPENDIX

Workshop Description:
The workshop was convened November 6-7, 2017 in Rockville, Maryland, USA. The aim of the workshop was to define the research agenda to facilitate the development of LA/ER products for both treatment and prevention in infants, children, adolescents and PLW and identify approaches for generating, in a timely manner, the data necessary for regulatory approval of LA/ER products for these groups. Participants were encouraged to consider the need for unique products and modes of drug delivery for infants and children, the safety of LA/ER products in pregnancy and during breastfeeding, and the potential role of LA/ER technologies for prevention and treatment of adolescents. Participants included clinicians, scientists, researchers, pharmacologists, pharmacists, drug regulatory specialists and community representatives. A complete list of participants is included.

The workshop began with a series of presentations by key experts on scientific and regulatory perspectives, global operations and implementation, the role of pharmacokinetic modelling, and challenges in drug development in relation to PLW, infants, children and adolescents. Participants were then assigned to one of four breakout groups, with discussion topics focusing on products for children, the inclusion of adolescents in clinical trials, managing PLW women on LA/ER products, and regulatory aspects relevant to these three key populations (table 1). Much of the data derived in Tables 2 and 3 were the result of consensus building within the breakout groups. Each group was moderated by two co-chairs and discussions were documented by a rapporteur. Breakout group discussions were also transcribed for the sole purpose of writing this manuscript. Groups were charged with developing a consensus statement identifying important gaps and recommendations for future work within their area. Key principles were later shared in a large group session, during which questions were raised. The complete content of all presentations is available online.[16]

Workshop Speakers:
Alison Agwu, Linda-Gail Bekker, Edmund Capparelli, Carl Dieffenbach, Magali Hickey, Bethany Stewart, Kimberly Struble, Heather Watts

Breakout Group Rapporteurs and co-Chairs:
Products for children session: Peter Havens (co-chair), Carolyn Bolton (co-chair), Marco Siccardi (Rapporteur); Pregnancy session: Elaine Abrams (co-chair), Shahin Lockman (co-chair), Polly Clayden (Rapporteur); Adolescent session: Sybil Hosek (co-chair), Kenneth Mayer (co-chair), Jen Cohen (Rapporteur); Regulatory session: Linda Lewis (co-chair), Ellen Chadwick (co-chair), Jeff Jacobson (Rapporteur).

Workshop Participants:
Elaine Abrams, Sharon Achilles, Adeola Adeyeye, Allison Agwu, Susannah Allison, Vikram Arya, Linda-Gail Bekker, Yodit Belew, Debra Birnkrant, Carolyn Bolton, Gina Brown, David Burger, Edmund Capparelli, Carmen Perez Casas, Ellen Chadwick, Nahida Chakhtoura, Yao Cheng, Su-Young Choi, David Claffey, Diana Clarke, Polly Clayden, Jen Cohen, Myron Cohen, Pablo Rojo Corneo, Carl Dieffenbach, Paul Domanico, Deborah Donnell, Terry Fenton, Charles Flexner, Susan Ford, Hanna George, Sheetal Ghelani, Pedro Goicochea, Sarit Golub, Charlie Gombar, Peter Havens, Trevor Hawkins, Rohan Hazra, Magali Hickey, Lisa Hightower-Weidman, Rodney Ho, Sybil Hosek, Jeffrey Jacobson, Patrick Jean-Phillipe, Sandee Juneja, Bill Kapogiannis, Andy Kaytes, Raphael Landovitz, Melissa Leavitt, Linda Lewis, Daniella Livnat, Shahin Lockman, Kenneth Mayer, Cindy McCoig, James McIntyre, Jane McKenzie-White, Peter Miele, Stephen Miller, Mark Mirochnick, Sharon Nachman, Parul Patel, Martina Penazzato, Natella Rakhmanina, Dianne Rausch, Theodore Ruel, Kim Scarsi, Marco Siccardi, Nirupama Sista, Bethany Stewart, Michael Stirratt, Kim Struble, Swindells Susan, Hedy Teppler, Claire Townsend, Lut Van Damme, Prabha Viswanathan, Jacqui Wambui, Melynda Watkins, Heather Watts, Peter Williams, Lei Zhu
